# Influenza Vaccination in the Face of Immune Exhaustion: Is Herd Immunity Effective for Protecting the Elderly?

**DOI:** 10.1155/2011/419216

**Published:** 2012-01-29

**Authors:** Pierre Olivier Lang, Dimitrios Samaras, Nikolaos Samaras, Sheila Govind, Richard Aspinall

**Affiliations:** ^1^Department of Internal Medicine, Rehabilitation and Geriatrics, Medical school and University Hospitals of Geneva, CH-1226 Thônex-Geneva, Switzerland; ^2^Translational Medicine Research Group, Cranfield Health, Cranfield University, Cranfield MK43 0AL, UK; ^3^Nutrition Unit, Medical school and University Hospitals of Geneva, CH-1205 Geneva, Switzerland

## Abstract

At the start of the 21st century, seasonal influenza virus infection is still a major public health concern across the world. The recent body of evidence confirms that trivalent inactivated influenza vaccines (TIVs) are not optimal within the population who account for approximately 90% of all influenza-related death: elderly and chronically ill individuals regardless of age. With the ever increasing aging of the world population and the recent fears of any pandemic influenza rife, great efforts and resources have been dedicated to developing more immunogenic vaccines and strategies for enhancing protection in these higher-risk groups. This paper describes the mechanisms that shape immune response at the extreme ages of life and how they have been taken into account to design more effective immunization strategies for these vulnerable populations. Furthermore, consideration will be given to how herd immunity may provide an effective strategy in preventing the burden of seasonal influenza infection within the aged population.

## 1. Introduction

Infants and the elderly share a high vulnerability to infections and therefore have specific immunization requirement [1]. Foremost amongst vaccine infectious preventable diseases is influenza virus infection. Worldwide influenza causes 3–5 million of severe cases per year resulting in 250,000–350,000 deaths [2]. Indeed, while influenza affects people of all ages, young children and older adults, suffering or not from medical comorbid conditions, are particularly vulnerable. This results in increased morbidity and mortality [2–4]. While children have the highest rates of seasonal influenza infection and illness [3, 4], mortality in the elderly is just the tip of the iceberg in terms of disease burden. Influenza infection can also act within this population as a trigger for functional decline, decompensation of medical comorbid conditions, and/or cardiovascular and neurovascular acute diseases, being thus contributory to excessive hospitalization, antibiotic prescriptions, and a considerable economic burden [5–10]. Indeed, early immune protection initially relies on maternal antibodies, and this makes infants to become vulnerable to infections within a short time frame if failure of development of adaptive immunity in order to confer sustained protection [1, 11]. In those who approach the end of the normal life expectancy, the age-related decline in immune function, usually termed immunosenescence, partly explains the inability to resist influenza virus. Moreover, the elderly not only have problems in dealing with new pathogens but also have difficulties in responding to pathogens that they previously overcome [11–13].

Vaccination is considered to be the cornerstone for preventing morbidity and mortality associated with influenza infection. Immunization programs are timed to optimize protection during an influenza season [2, 14]. Current vaccines contain 15 *μ*g of the hemagglutinin (HA) of an A/H1N1, A/H3N2, and B strain and are given to induce serum anti-HA antibody for prevention of subsequent infection and illness from natural influenza. Trivalent inactivated influenza vaccines (TIV) are widely used with approximately 300 million doses produced each year [9]. However, the ability of TIV to induce effective protection is related to age. With an efficacy between 70% and 90% in individuals ranging in age from 7 to 65 years when the vaccine and circulating virus are antigenically similar [14–16]; the picture at the extreme ages of life is completely different [4, 17, 18]. TIV is poorly immunogenic in young children, with an efficacy of only 59% in children older than 2 years of age [16, 19, 20] and only of 30% to 40% at best for those over 65 years of age [9, 11, 15]. However, in the elderly, few placebo-controlled randomized clinical trials of influenza vaccine have been done [16] and influenza vaccine effectiveness estimates mainly are derived from observational studies typically using data from research databases or health care utilization data systems [9, 17]. The largest and best-designed placebo-controlled trial was done by Govaert et al. [18]. After stratifying by age, the estimated vaccine efficacy was of 57% in people aged 60–69 years and 23% in volunteers aged 70 years or older.

This paper describes the mechanisms that shape immune response at the extreme ages of life and how they have been taken into account to design more effective immunization strategies for these vulnerable populations. Furthermore, consideration will be given to how herd immunity may provide an effective strategy in preventing the burden of seasonal influenza infection within the aged population.

## 2. Improving the Ability of Both Immature and Senescent Immune System to Respond to Influenza Vaccination

Stimulation of a primary immune response following vaccination involves the activation of innate immunity followed by the activation and differentiation of naive lymphocytes by viral antigen and their differentiation into memory T and B cells and antibody-secreting plasma B cells. Long-term immunity is assured by memory cells in the blood and lymph nodes, as well as by long-lived plasma cells and memory T cells in the bone marrow [12]. High levels of neutralizing antibody are required for protection against pathogen both in early and late life [11]. Thus, as current TIV do not offer optimal protection at the extreme ages of life, great efforts and resources have been dedicated both to infant and aged adults immunization [1, 9, 11, 19] with notable impacts on morbidity and mortality in youngsters [1, 20, 21].

### 2.1. Circumventing the Limitation of Vaccine Response in Early Life

New adjuvants and/or delivery systems that increase activation of the immature innate and adaptive immunities and plasma-cell differentiation have been suggested. However, defining the earliest age at which specific vaccine antigens can efficiently prime neonatal B and T cells of the appropriate phenotype was the challenge of vaccination in early life. Thus, although intranasal live attenuated influenza vaccine has an efficacy of 69 to 95% in children of 2 to 7 years of age, this vaccine cannot be used under the age of 5 years because of the increased risk of adverse events [22, 23]. Adjuvanted TIV (ATIV) using MF59 induces greater immune response than TIV in children 6 to 36 months of age [24, 25]. Recently, it has been demonstrated that the MF59-adjuvant vaccine was efficacious against laboratory-confirmed influenza caused by all circulating viral strains in healthy children 6 to less than 72 months of age with an efficacy rate of 86% and with higher efficacy against vaccine-matched strains (89%) [20]. In contrast, the respective efficacy rates for TIV in those children who had not previously been vaccinated against influenza were 43% and 45%, resulting in relative efficacy rates of 75% and 80% for ATIV as compared to TIV.

### 2.2. Circumventing the Limitation of Vaccine Response in Later Life

While the adaptive immunity in infants is readily amenable to enhancement through vaccination [1, 11], the age-associated atrophy of the haematopoietic tissue and primary lymphoid organs are important limiting factors [13, 26]. Indeed, the accumulation of fat in the thymus and the bone marrow is associated with decreased production and export of new B and T cells resulting in an immune system gradually getting weaker with advancing age [8, 27]. Thus, older individuals have fewer naive cells, more memory cells, and an ever increasing number of senescent cells which are known to exert regulatory role *in vivo* [8, 9, 12, 28–30]. Novel vaccine formulations with increased doses of antigen or new adjuvants have been developed in order to enhance vaccine immunogenicity by providing a stronger stimulus [5, 9, 19, 29, 31, 32]. Potent adjuvant can potentially improve immunogenicity through increasing response to toll-like receptor-mediated signaling. Virosome, MF59, AS03, and other adjuvants or intradermal or higher-dose vaccines (15 *μ*g of HA versus 30 and 60 *μ*g) indeed modestly improve immunogenicity, but do not restore immunogenicity to that of younger individuals, suggesting the existence of other crucial factors [1]. Moreover, it is not yet clear how this will translate into protection against all usual influenza-associated clinical endpoints, though for the moment an increased vaccine reactogenicity and higher side effects were measured [19]. Moreover, all these alternate strategies mainly focus on the initial steps of the immunological process of the vaccine response, and therefore they over stimulate the naive cell pool that are the most reduced during the immunosenescence process [12], without any consideration to the pool that affect the most immune response: senescent cells [30, 33]. Thus, whether vaccine prevention can be improved in the aged adult population by developing novel vaccine or would need strategies to slow or reverse the immune senescence process is still a challenging and debating issue [1, 27, 33].

### 2.3. Strategies for Reversing the Immune Senescence Process

Thymic atrophy is the key element of changes that occur in the age-associated decline in immunity [13]. Thus, different ways have been explored regarding how best to rejuvenate better the peripheral T-cell pool and delay or reverse the immune decline [9, 27, 29]. Thymic rejuvenation techniques are still in early stages of clinical trials and can be categorized in to the 3R's of rejuvenation: restoration, replacement, and reprogramming [27]. Restoration strategy aims to maintain a normal thymic environment by using cytokines, growth hormone, sex-steroids, growth factors, and nutrients. Replacement strategies aim to restore immune function lost by several techniques including the transfusion of autologous blood derived from individuals during their early life and transfused when they are much older or alternatively the transfer of *ex vivo* generated naïve T cells into individuals with defective thymopoiesis. In addition, the most “revolutionary” treatment could be based on reprogramming the immune system, for example by pharmacologic approaches that enhance telomerase as a possible means for the prevention or retardation of replicative senescent cells [9, 29, 34]. It has also been proposed to physically remove from the circulation and/or precipitate the apoptosis of senescent cells with the hope of inducing the homeostatic expansion of more functional population of memory T cells [9].

### 2.4. Toward a Changing of the Immunization Strategy Against Influenza?

Immunization strategies are crucially important in preventing influenza infection and directly protecting vulnerable population from influenza virus. However, as previously described, the efficacy of the influenza vaccine mainly depends on the quality of the immune system that is stimulated, and this even when a stronger stimulus is provided. Paradoxically, the global success of mass immunization strategies contrasts with the largely recognized failure of strategies targeting individuals at increased risk of complications, whether from underlying disease, treatment, or age [1]. Thus, in contrast with the success of childhood vaccine programmes [35], vaccine coverage rates concerning the most common vaccine preventable infectious diseases are still very low within the adult population and this even in the higher-risk and older age groups [28]. Despite strong and widespread recommendations [14, 36], underlying structural, logistical, economic, cultural, and political issues contribute to this outcomes [37–40]. The striking imbalance between children and aged adults, not only in terms of vaccine coverage rate and vaccine effectiveness, but also in the epidemiology and influenza-associated burden of illness, has led some authors to recommend routine seasonal vaccination to directly protect children and indirectly the entire population [3, 4, 28, 33, 41–44].

## 3. When Vaccinated Children Can Protect from Influenza Nonimmunized Adults

### 3.1. Herd Immunity: Definition of a Concept

Herd immunity describes a form of “immunity” that occurs when the vaccination of a significant portion of the population provides a measure of protection for individuals who are not vaccinated [45, 46]. Herd immunity theory proposes that, in contagious diseases that are transmitted from individual to individual (i.e., influenza) and/or for which human is an important reservoir (i.e., diphtheria), the chain of infection is likely to be disrupted when large numbers of a population are immunized. This has the effect of increasing the level of population (or herd) immunity and reducing the likelihood that susceptible individuals (i.e., not or incompletely vaccinated or those in whom vaccination is contraindicated or considered as less or not-effective) will be infected [28, 33].

### 3.2. The Quasiexperimental Demonstration of Its Efficacy in Preventing Influenza Infection

Vaccination programs currently recommend that aged adults (United States—US: ≥ 50 years of age [47]; Austria, Germany, Hungary, Russia: ≥ 60 years of age and most of other European countries: ≥ 65 years of age [36]) and those suffering from chronic comorbid medical conditions should be vaccinated against influenza each year [47]. A quasiexperimental demonstration of the impact of herd immunity on controlling influenza has resulted from the retrospective analysis of the Japanese experience [44, 48, 49]. This country was the only one that has based its policy on a strategy of vaccinating schoolchildren with the aim to protect children and reduce the rate of transmission within the community and particularly in higher-risk groups. Unfortunately, assessments of the effectiveness of the programme were not focused on the higher-risk population, and the methods initially used to assess morbidity in schoolchildren were insufficiently sensitive to demonstrate any beneficial effect that could carry on after the discontinuation of the programme. Retrospectively, Reichert et al. has observed that the mandatory vaccination program initiated in 1962 significantly decreased excess mortality from seasonal influenza and invasive pneumococcal disease in adults and older adults [44]. The authors analyzed the monthly rates of death from all causes and death attributed to pneumonia and influenza for both Japan and US. While the excess of mortality was highly correlated in each country, and these rates were nearly constant over time in the US, the initiation of the Japanese vaccination programme significantly dropped the excess of mortality from values corresponding to three to four time those in the US. The law relaxed in 1987 and was repealed in 1994, which was subsequently followed by declined vaccination rates, a drop in population immunization levels, and a new increase in community deaths from influenza and invasive pneumococcal diseases, with an increasing tendency of the excess mortality in the 45–64 years and 65 years or over age groups [50, 51]. Excess mortality mainly concerned persons with chronic comorbid medical conditions (i.e., chronic obstructive pulmonary disease (COPD), heart and/or cerebrovascular diseases, cancers, and renal failure) accounting respectively for approximately 20–50%, 20–40%, 20%, 5%, and 2% of all the excess mortality [51]. Finally, it was estimated that the vaccination of Japanese children prevented about 37,000 to 49,000 deaths per year mainly among older person, or about 1 death for every 420 children vaccinated.

## 4. Is Herd Immunity a Sustainable Approach in Our Ever Networking and Greying World

The beneficial effects for herd immunity are now well documented. This is not only for influenza infection as previously demonstrated but also effective for pneumococcal diseases, measles, pertussis, and diphtheria prevention [28, 33]. However, one of the major flaws in the argument that herd immunity would be beneficial in order to avoid the problems associated with infection in the older adults is that this would be effective if this population remained within the community. However, this is not the reality, and individuals now travel more and more than either their grandparents or parents [52]. The world is now more closely networked than before, and the increased amount of air travel means that the spread of any pathogen across the globe can occur within hours as demonstrated by recent spread of the avian influenza A H5N1 and swine influenza A H1N1 strains [1]. The elderly are increasingly part of this trend [33]. Moreover, vaccine responses are unfortunately not uniform worldwide, and variable immune responses depending on environmental and host genetic factors are elicited [1, 53, 54].

The second major flow is that the sufficient mass of the population to vaccinate in order to reach the herd immunity threshold is questionable in our ever increasing aging population [33]. The united nations have coined the term “demographic” transition to describe over the last 5 decades the tripling of the number of individuals older than 65 years [55]. Currently, the growth rate of the older part of the global population is significantly higher than the global population. By 2025–2050, projections indicate that the population aged over 60 will be growing 3.5 times as rapidly as the total population. Within the next 40 years, the European rural development project (http://www.iiasa.ac.at/Research/ERD/) suggests that there will be about 90 million people under 14 years of age and around 264 million people over the age of 65. Europe may currently lead the world with the highest proportions of older adults, but this may not last much longer. By 2050, nearly four fifths of the world older population will be living in the less developed regions of the world [33]. The optimism created by living longer must however be balanced by the reality of the burden placed on the society, the medical and social welfare services by the increased number of older individuals [56]. Indeed, we cannot escape the simple fact that human ageing is inextricably linked with an increasing incidence of chronic disorders [57]; conditions that further impinge the immune system by inducing a chronic low grade inflammation [7, 28, 58, 59]. Moreover, when immunization programmes are nearly completely childhood centered, as they are considered by most of the industrialized countries, careful consideration must be given also to the herd immunity potential deleterious effects [60]. These perspectives thus reinforce the importance of maintaining high vaccination coverage rates not only in children but also optimizing vaccine uptake during adulthood [43]. Concerning specifically influenza, it has been well demonstrated that vaccine coverage rates within adult population are still worst than those measured in the aged population, and this even in the high-risk groups [61]. However, the European guidelines have not found enough evidence to target other groups [62], but caregivers, health care workers (including ancillary staff), pregnant women, and healthy children (younger than 16 years) are also strongly advised to receive influenza vaccinations [63]. These recommendations could be easily extended to all people who live with or care for older adults as it is now advised by ACIP in the USA [64]. However, as demonstrated by Michiels et al. in a recent review of the evidence on the effectiveness of TIV in these different other target groups, many limitations make the conclusion in the present guidelines questionable [62]. Indeed, the achievement of an accurate assessment of vaccine benefits is still fraught with considerable methodological and epidemiological challenges. Thus, while TIV shows efficacy in healthy adults and children 6 years old or over [21, 62, 65], inconsistent results are found in studies among children younger than 6 years old, individuals with chronic-comorbid conditions and healthcare workers, which might be completely explained by biases. However, the authors confirmed that vaccination of pregnant women might be beneficial for protecting their newborns, and vaccination of children might be protective in the nonimmunized of all ages living in the same community [5, 62, 66, 67].

## 5. Conclusion

Seasonal influenza virus infection remains a major public health concern across the world, and the recent body of evidence confirms that TIVs are optimal only in healthy adults and children. Through the effect of herd immunity, vaccination of pregnant women and children is also protective for newborns and nonimmunized individuals of all ages. These evidences have led some to promote yearly childhood vaccination to directly protect children and indirectly the entire population, including even the higher-risk groups. However, the current demographic shift and the sufficient mass of the population to vaccinate in order to reach the herd immunity threshold make this approach questionable. A potential alternative model could be to move into a broader thinking that shifts the emphasis toward a more balanced approach across the life span by targeting other healthy groups as health care professionals and all adults who live and/or care for older adults. This, however, implies to better understand how to break down the public, cultural, societal, and political barriers to immunization and counter antivaccination movement that highly contributes to reduce the acceptance of influenza vaccine within these populations. As a consequence, another challenge will be also to produce good quality and publicly founded data in order to support new vaccine formulations with strong evidence. Thus, comparing this novel technology in well-designed and head-to-head clinical trials with current formulations would be a competitive alternative. This could limit the considerable methodological and epidemiological biases that impinged the accurate assessment of inactivated influenza vaccine benefits.
